# Anatomical Tibial Tunnel Vector in Pull-Out Repair for a Medial Meniscal Posterior Root Tear (MMPRT) and Its Clinical Potential

**DOI:** 10.7759/cureus.89122

**Published:** 2025-07-31

**Authors:** Kensaku Abe, Toshio Morinaga, Megumu Kawai, Kazuki Hanashima, Masaaki Akasaka, Junji Sakashita

**Affiliations:** 1 Department of Orthopaedic Surgery, Keiju Medical Center, Nanao, JPN; 2 Division of Radiology, Keiju Medical Center, Nanao, JPN

**Keywords:** anatomical, high tibial osteotomy, medial meniscus, posterior root tear, pull-out repair, tibial tunnel

## Abstract

Introduction

This study focuses on the tibial tunnel in the pull-out repair of medial meniscus posterior root tears (MMPRTs), with attention to the distal hole as the anatomical vector. Little research has explored this aspect, as previous studies have mainly emphasized the proximal hole as the attachment point.

Methods

The anatomical vector was identified by analyzing magnetic resonance images of 84 healthy knees. Clinical nine cases that underwent pull-out repair using the anatomical tibial tunnel (AT) with open wedge high tibial osteotomy (OWHTO) were evaluated to investigate the interference between plate screws and AT, and longitudinal changes in tibial tunnel diameter.

Results

Almost all distal holes of the tibial tunnels were plotted on the anterolateral surface of the tibia. The distal hole of the anatomical vector was more lateral in the ‘body mass index of more than 22 kg/m2’ and ‘currently with sports’ groups. In only one clinical case, the AT interfered with the proximal anterior screw. The tibial tunnel diameter was significantly reduced in the AT group compared with the medial tibial tunnel (MT).

Conclusions

The anatomical vector pointed to the anterolateral surface of the tibia and tended to point outward due to potential overload. Compared with the MT, the AT is expected to reduce the killer turn of the posterior root, promoting early tibial tunnel filling, and improving interference issues with screws in OWHTO. The AT can be an option for pull-out repair of MMPRTs.

## Introduction

Recently, as the importance of the posterior root of the medial meniscus (MM) has been particularly recognized, surgeons have been attempting to repair MM posterior root tears (MMPRTs) [1]. MM posterior root (MMPR) repair can reduce the excessive tibiofemoral contact pressure caused by MMPRT by anchoring the MMPR and MM horn [2]. Several arthroscopic repair techniques, such as transtibial pull-out repair and suture anchor-dependent repair, show more favorable clinical outcomes than conservative treatments in patients with MMPRTs [3,4]. In arthroscopic MMPR repairs, the accurate positioning of the tibial tunnel aperture seems to be critical in restoring meniscal function following transtibial pull-out repair [3]. The anatomical repair produced a near-intact contact area and minimally increased the mean and peak contact pressures compared with those of an intact knee [5]. An anatomical placement of an MMPR attachment is considered critical in transtibial pull-out repair of MMPRTs [6], and anatomical reduction of the meniscus root attachment and restoration of the native root position recovers the integrity of the knee and prevents the progression of this natural history [7]. Furthermore, Moatshe G et al. have reported that the anatomical placement of the MMPR/MM horn is considered necessary for obtaining good clinical outcomes in patients with MMPRT following repair [8]. However, although some reports have reported that tensile forces applied to the MM posterior horn and repaired tissue vary in the tibial tunnel position, and the suture fixation for each patient must be optimized [9], few reports have described the distal hole of the transtibial tunnel. In the past, methods mainly introduced the lateral tunnel [10-13]. Even in the few reports of the lateral tibial tunnel, the MMPR is only pulled out to the lateral side, and there is no mention of where to pull out biomechanically. In recent years, the medial tunnel has tended to be preferred [14], possibly due to its technical convenience. From a surgical standpoint, the medial side provides easier access to the tibia because there is no intervening muscle tissue, only subcutaneous tissue, making tunnel creation more straightforward. Furthermore, when combined with open-wedge high tibial osteotomy (OWHTO), the same incision can be used for both procedures, reducing invasiveness. These considerations may contribute to the increased preference for the medial tunnel. However, a distal hole in the transtibial tunnel was created at the medial side of the tibia, a killer turn of the MMPR was often observed, and there was concern about rupture due to friction in the long term. Kwon SW et al. compared medial and lateral tibial tunnels in terms of anatomical accuracy and reported that the lateral tibial tunnel more consistently reached the intended attachment point of the MMPR [14]. However, we were unable to identify studies directly comparing these techniques in terms of biomechanical properties or clinical outcomes. Moreover, the position of screw interference when combined with OWHTO in the medial transtibial tunnel is a concern.

Therefore, in this study, we first aimed to evaluate the anatomical vector of the MMPR, representing its three-dimensional orientation, using MRI analysis. As a secondary objective, we investigated the individual factors associated with variations in this vector. Based on its direction, we defined the anatomical tibial tunnel (AT) as a tibial tunnel aligned with the anatomical vector. Finally, to assess the potential clinical applicability of the AT, we compared postoperative outcomes of transtibial pull-out repairs using the AT with those using the conventional medial tibial tunnel in patients undergoing concomitant OWHTO.

## Materials and methods

Examination of the anatomical vector of the MMPR

Magnetic resonance imaging (MRI) was performed on 84 healthy knees without any history of disease or injury at our institution. The mean age of the subjects was 28.2 ± 5.0 years (range, 21−39 years), the mean height was 165.3 ± 9.3 cm (range, 148−186 cm), the mean weight was 62.3 ± 14.3 kg (range, 38−112 kg), and the mean body mass index (BMI) was 22.6 ± 3.8 kg/m² (range, 16.9−37.8 kg/m²).

MRI scans were acquired using a 3.0-T scanner. Axial T2-weighted images with a slice thickness of 1 mm were obtained to evaluate the MMPR. Sagittal images were reconstructed from the axial dataset using multiplanar reconstruction techniques for further analysis.

**Figure 1:**
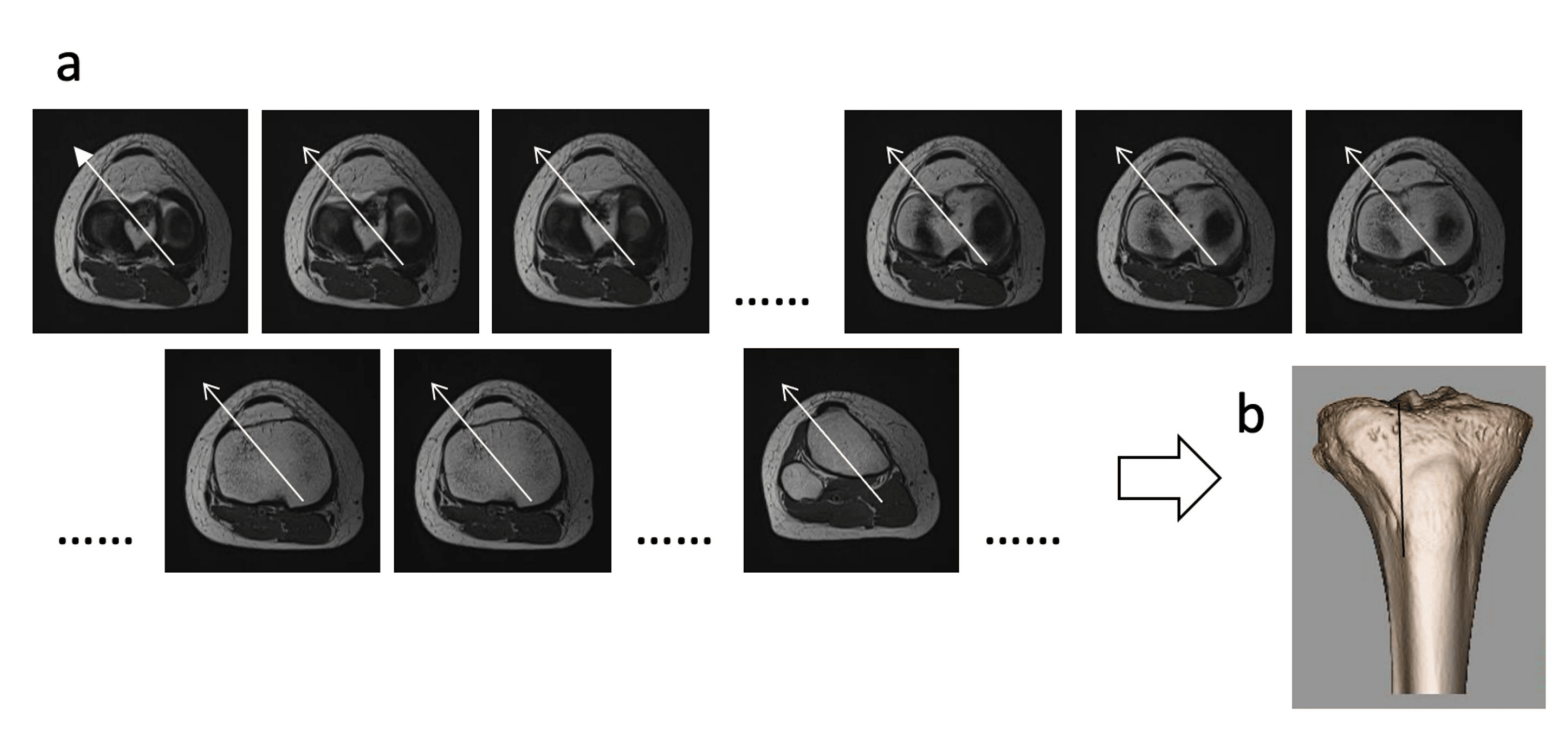
Determination of the anatomical vector of the MMPR on axial MRI slices (a) Axial MRI slices used to identify the anatomical vector of the medial meniscus posterior root (MMPR). Based on the first slice (arrowhead), the direction of the anatomical vector was marked with arrows and copied distally through sequential slices. (b) A 3D reconstructed image showing the anatomical vector projected onto the tibial plateau in the axial plane.

To define the anatomical vector of the MMPR, we first identified the axial slice in which the MMPR was most clearly visible. As shown in Figure 1, a line bisecting the medial and lateral edges of the MMPR was drawn on this axial image, and the line was extended distally in parallel to identify its intersection with the tibial cortex.

**Figure 2:**
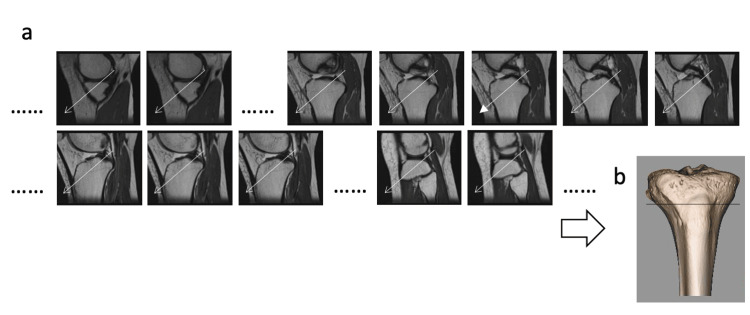
Determination of the anatomical vector of the MMPR on sagittal MRI slices (a) Sagittal MRI slices used to identify the anatomical vector of the MMPR. Based on the fifth slice (arrowhead), the direction was marked with arrows and extended medially and laterally across the slices. (b) A 3D reconstructed image showing the anatomical vector projected onto the tibial plateau in the sagittal plane. MMPR: medial meniscus posterior root

**Figure 3:**
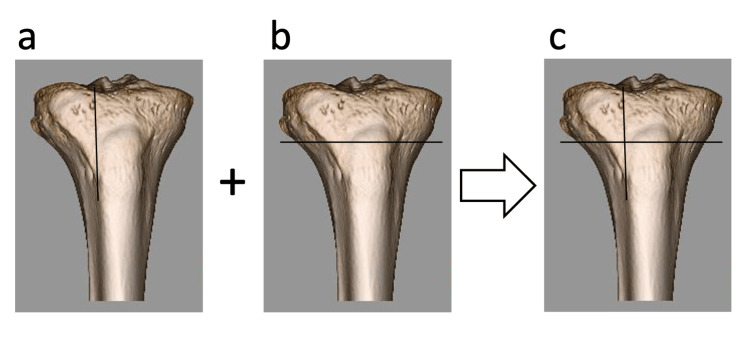
The procedure identified the anatomical vector of the medial meniscus posterior root (MMPR) (a) Axial image corresponding to Figure 1, showing the vertical line of the anatomical vector. (b) Sagittal image corresponding to Figure 2, showing the horizontal line of the anatomical vector. (c) The intersection of the vertical and horizontal lines from (a) and (b) defines the destination of the anatomical vector of the MMPR.

Similarly, in the sagittal plane, the MMPR was vertically bisected as shown in Figure 2, and this line was extended mediolaterally to determine its intersection with the medial and lateral surfaces of the tibial cortex. The intersection point of these two lines was defined as the distal exit of the anatomical vector for that subject (Figure 3).

The distal exit point of this vector at the tibial cortex was plotted on a 3D-CT image of the tibia. For subsequent analysis, these points were divided into four quadrants (upper/lower and medial/lateral) using orthogonal vertical and horizontal reference lines. These lines were manually set to approximately divide the distribution of points into comparable groups, as no anatomical or radiographic reference point was available.

To investigate potential factors influencing vector orientation, multivariate logistic regression analysis was performed. The following variables were included: gender (male, 37; female, 47), age (≥28 years, 39; <28 years, 45), height (≥165 cm, 45; <165 cm, 39), weight (≥62 kg, 37; <62 kg, 47), BMI (>22 kg/m², 41; ≤22 kg/m², 43), current participation in sports (yes, 20; no, 64), history of sports activity during adolescence or adulthood (yes, 71; no, 13), history of injury to the contralateral knee (yes, 10; no, 74), and laterality of the imaged knee (dominant, 37; non-dominant, 47). Current participation in sports was defined as regular engagement in recreational or competitive physical activities at least once per week within the past six months. Subjects with unclear history or inconsistent activity were excluded from this classification.

Evaluation of the clinical cases after pull-out repair using AT with OWHTO

Nine knees underwent OWHTO, MM centralization, and pull-out repair using AT (mean age, 66.1 years; female, 7; BMI, 26.6 kg/m²). Postoperative computed tomography (CT) was used to investigate the interference between the plate screw in OWHTO and the AT. Furthermore, we measured that the distance of the distal hole was away from the ideal area obtained in the subsection "Examination of the anatomical vector of the MMPR". We also investigated the longitudinal changes in tibial tunnel diameter in cases where pull-out repair was performed in the medial tibial tunnel (MT) and the AT without the use of bone-formation-promoting agents like teriparatide. We were able to follow up with 5 cases each at multiple time points, including the 1-year mark, for the duration of the study (AT (mean age, 61.2 years; female, 4; BMI, 27.7 kg/m²), MT (mean age, 67.3 years; female, 4; BMI, 27.7 kg/m²)). The tibial tunnel diameter was defined as the average length measured in the axial, sagittal, and coronal images of the CT. The tibial tunnel created during the surgery was made using either a 4.0 mm or 4.5 mm drill (it is unknown which size was used in each case). For the five cases conducted in the MT group, evaluations were performed at one week, three months, six months, and one year after the surgery. As for the five cases in the AT group, three cases were evaluated at the same time points as the MT group, while two cases were evaluated at one week, three weeks, six months, and one year after the surgery. During the statistical analysis, the time points were simplified as follows: one week, three weeks to three months, six months, and one year. When conducting statistical analysis, the tibial tunnel diameter at 1 week was taken as a reference (ratio = 1.0) for comparative analysis.

Statistical analysis

Statistical analysis was performed using EZR (Saitama Medical Center, Jichi Medical University, Saitama, Japan), which is a graphical user interface for R (The R Foundation for Statistical Computing, Vienna, Austria). More precisely, it is a modified version of R Commander designed to add statistical functions frequently used in biostatistics [15]. For analysis of factors related to anatomical vector orientation, multivariate logistic regression analysis was conducted. To evaluate changes in tibial tunnel diameter over time, the Mann-Whitney U test was used for between-group comparisons, and repeated measures analysis of variance (ANOVA) was used to assess longitudinal changes. P-values of less than 0.05 were considered statistically significant.

Statement and informed consent

This study was approved by the Institutional Review Board of Keiju Medical Center (Ref. No. 2021-1-1). Informed consent to be included in this study and for the publication of this study and any accompanying images was obtained from all patients and subjects with healthy knees. All experiments were performed in accordance with the Declaration of Helsinki.

## Results

Examination of the anatomical vector of the MMPR

**Figure 4:**
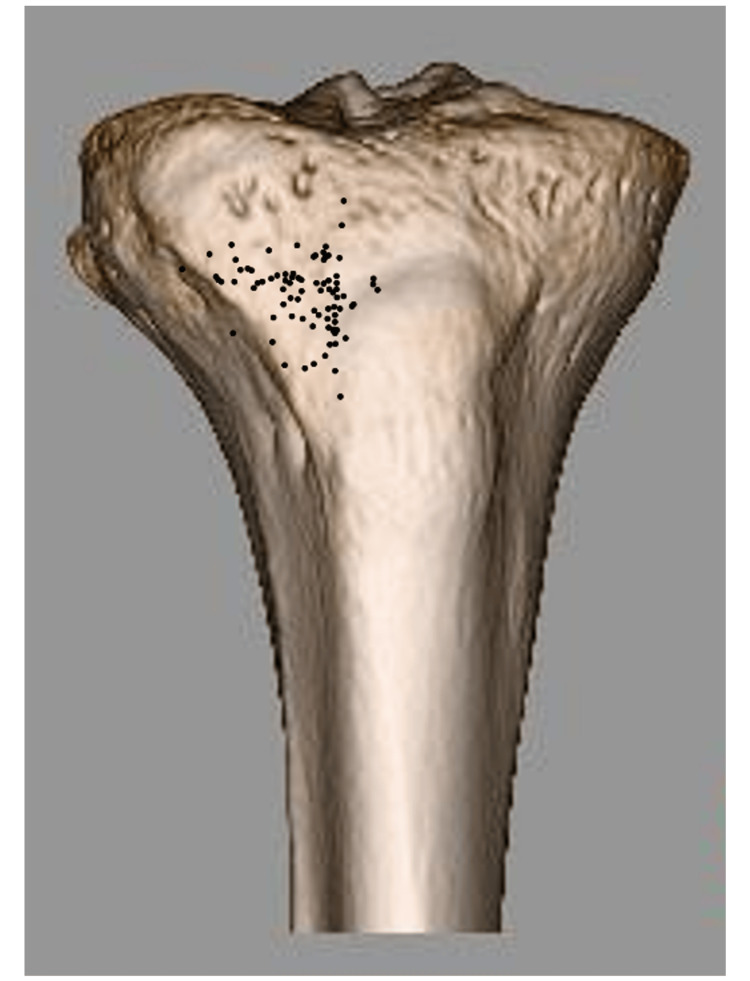
Distribution of anatomical vector destinations of the medial meniscus posterior root (MMPR) Each black dot represents the distal destination point of the anatomical vector of the medial meniscus posterior root on the tibia, based on MRI analysis.

Almost all distal holes of the tibial tunnels were plotted on the anterolateral surface of the tibia (within the triangle surrounding the medial edge of the tibialis anterior attachment and the lateral edge of the tibial tuberosity) (Figure 4).

**Figure 5:**
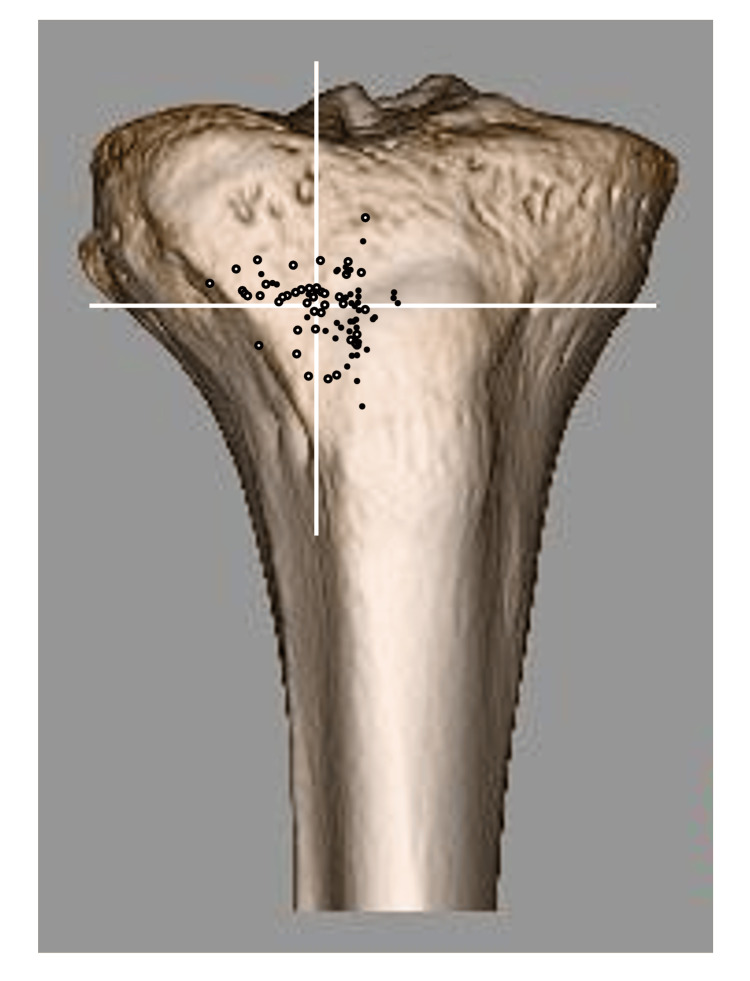
Distribution of anatomical vector destinations by BMI group Black dots and white dots indicate the destinations of the anatomical vector in the low BMI group and the high BMI group, respectively. White vertical and horizontal lines divide the medial and lateral, and the upper and lower compartments, respectively.

**Figure 6:**
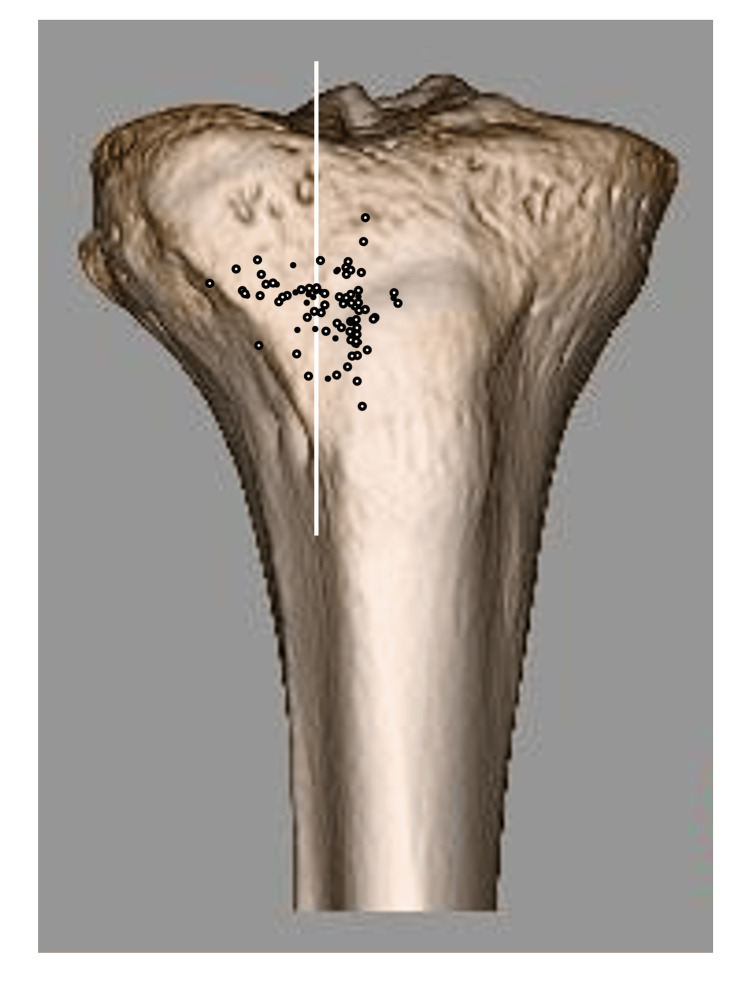
Distribution of anatomical vector destinations by current sports participation group Black dots and white dots indicate the destinations of the anatomical vector in the group currently participating in sports and not currently participating in sports, respectively. The white vertical line divides the medial and lateral compartments.

In multivariate logistic regression analysis, the anatomical vector significantly pointed to the upper and lateral sides in the group with a high BMI (>22 kg/m2) (p = 0.04 and p < 0.01, respectively) and the lateral side in the group of people currently participating in sports (p < 0.01). The initial analysis showed that the anatomical vector tended to point to the medial side in the group with a history of sports; however, the second analysis with backward elimination showed no significant difference (Figures 5, 6, and Tables 1, 2).

**Table 1:** Correlation of the anatomical vector of the tibial tunnel with each factor Values are presented as the number of knees in each category. Multivariate logistic regression analysis was performed to examine the association between the tibial tunnel direction and each variable. Odds ratios (ORs), 95% confidence intervals (CIs), and p-values are shown. *p < 0.05 was considered statistically significant. OR: odds ratio; CI: confidence interval; N/A: not applicable

		Compartment		Multivariate logistic regression			Compartment		Multivariate logistic regression		
		Upper	Lower	OR	95% CI	P value	Medial	Lateral	OR	95% CI	P value
Gender	Male/female	26/29	11/18	2.26	0.42−12.0	0.34	13/31	24/16	1.33	0.24−7.39	0.29
Age (years)	≥28/<28	29/26	10/19	2.39	0.82−6.98	0.11	20/24	19/21	0.85	0.26−2.78	0.79
Height (cm)	≥165/<165	30/25	15/14	0.66	0.13−3.24	0.61	17/27	28/12	0.22	0.04−1.23	0.08
Weight (kg)	≥62/<62	26/29	11/18	0.34	0.06−1.78	0.20	10/34	27/13	0.36	0.07−1.88	0.22
BMI	>22/≤22	31/24	10/19	4.07	1.11−14.9	0.04*	12/32	29/11	0.16	0.04−0.63	<0.01*
Currently with sports^#1^	Yes/No	14/41	6/23	0.80	0.23−2.81	0.73	5/39	15/25	8.32	0.85−37.5	<0.01*
Sports history	Yes/No	47/8	24/5	0.74	0.16−3.41	0.70	38/6	33/7	0.14	0.02−0.79	0.03*
Injury history^#2^	Yes/No	10/45	0/29	<0.01	0−N/A (>1)	0.99	4/40	6/34	0.79	0.13−4.68	0.79
Dominant foot^#3^	Yes/No	25/30	12/17	1.40	0.51−3.90	0.51	21/23	16/24	1.65	0.52−5.20	0.39
^#1^currently participating in sports, ^#2^injury history of the reverse knee, ^#3^whether the photographed knee was the dominant one. OR, odds ratio; CI, confidential interval; N/A, not applicable, * p < 0.05											

**Table 2:** Correlation of the anatomical vector of the tibial tunnel with each factor after backward elimination Multivariate logistic regression analysis using the backward elimination method was performed for variables that showed significance in Table 1 to determine the independent association with the tibial tunnel direction (medial vs lateral). Odds ratios (ORs), 95% confidence intervals (CIs), and p-values are shown. *p < 0.05 was considered statistically significant. OR: odds ratio; CI: confidence interval; N/A: not applicable

		Compartment		Multivariate logistic regression		
		Medial	Lateral	OR	95% CI	P value
BMI	>22/≤22	12/32	29/11	0.11	0.04−0.32	<0.01*
Currently with sports^#1^	Yes/No	5/39	15/25	8.53	2.17−33.6	<0.01*
Sports history^#2^	Yes/No	38/6	33/7	0.44	0.10−1083	0.26
^#1^currently participating in sports, ^#2^injury history of the reverse knee. OR, odds ratio; CI, confidential interval; N/A, not applicable, * p < 0.05						

Evaluation of the clinical cases after pull-out repair using AT with OWHTO

**Figure 7:**
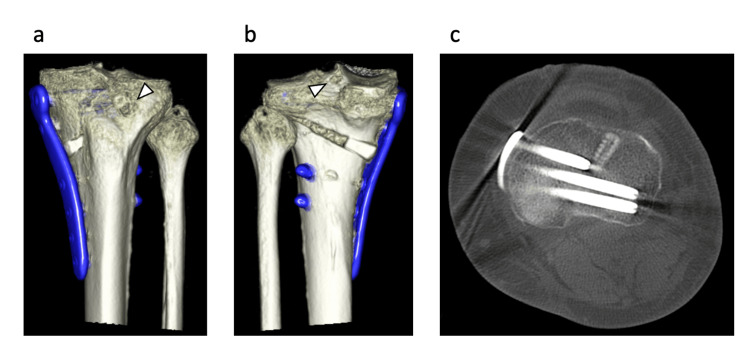
Our clinical case that underwent open-wedge high tibial osteotomy (OWHTO) and pull-out repair using the anatomical tibial tunnel (AT) (a) Three-dimensional CT image showing the distal hole of the AT, indicated by the arrowhead. (b) Three-dimensional CT image showing the proximal hole of the AT (at the medial meniscus posterior root attachment site), indicated by the arrowhead. (c) Axial CT image demonstrating that the anatomical tibial tunnel intersects with the anterior screw.

Only one case had interference between an AT and anterior proximal screw at 58.5% point (38 mm) of the length of the center proximal screw (65 mm) (Figure 7).

**Figure 8:**
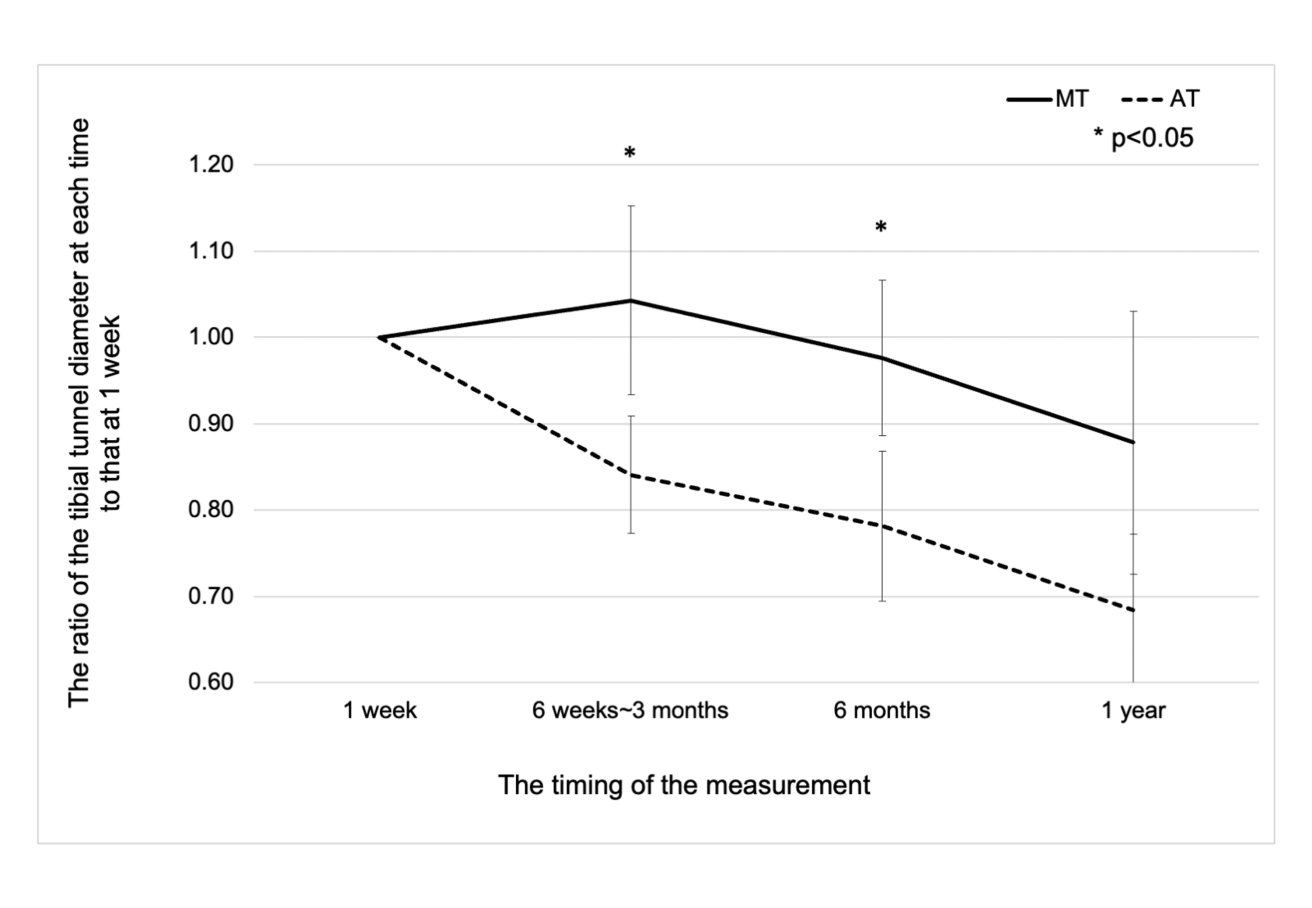
Graph representing the analysis of the tibial tunnel diameter with the first week as the reference It compares the medial tibial tunnel (MT) and the anatomical tibial tunnel (AT), where the solid line represents MT and the dashed line represents AT. The asterisk (*) indicates points where the Mann-Whitney U test was performed for the comparison between MT and AT, with p < 0.05.

The distal holes are located 9.91±2.71 mm (range, 6.24−14.5 mm) proximal to the ideal area. The tibial tunnel diameter at 1 week, 6 weeks to 3 months, 6 months, and 1 year was 4.09±0.16 mm, 4.27±0.54 mm, 3.99±0.38 mm, and 3.60±0.68 mm, respectively, in the MT group, and 4.43±0.54 mm, 3.73±0.63 mm, 3.47±0.68 mm, and 3.05±0.68 mm, respectively, in the AT group. In the analysis with the first week as the reference, the Mann-Whitney U test showed significant differences at 6 weeks to 3 months and 6 months (p=0.012, 0.012, respectively), but no significant difference at 1 year (p=0.059). The repeated measures analysis of variance revealed a significant difference between the MT and AT groups (p=0.007) (Figure 8).

## Discussion

Previous studies evaluating the tibial tunnel in MMPR pull-out repair have primarily focused on the proximal tunnel aperture located at the tibial plateau surface [16-18]. However, the anatomical path of the MM fibers, which travel obliquely from the MM horn to its tibial attachment, suggests that the direction and position of the distal tunnel hole, where the suture exits the tibial cortex, may also play a critical role in reproducing native fiber tension and minimizing graft abrasion. This study is the first to define the anatomical vector of the MMPR based on MRI and to use this vector to guide the location and direction of the tibial tunnel in a pull-out repair technique. We introduced the term anatomical tibial tunnel (AT) to describe a tibial tunnel aligned with the individual’s anatomical MMPR vector and evaluated its potential utility from anatomical and clinical perspectives.

The anatomical vector of the MMPR was consistently directed toward the anterolateral surface of the tibia, typically within the triangular region bordered by the medial edge of the tibialis anterior attachment and the lateral edge of the tibial tuberosity. Subgroup analysis showed that this vector tended to shift further laterally in individuals with higher BMI and those currently participating in sports. While the association with high BMI aligns with previous reports linking BMI to MMPRTs [19,20], the trend in sports-active individuals contrasts with earlier findings that MMPRT patients generally exhibit lower sports activity levels [19]. This discrepancy may reflect differences in study focus, vector orientation vs. activity level, and suggests that lateral orientation may result from biomechanical overload. However, variations in individual knee anatomy, such as tibial plateau morphology or coronal alignment, may limit the ability to consistently align the tunnel with the anatomical vector, which warrants further investigation.

**Figure 9:**
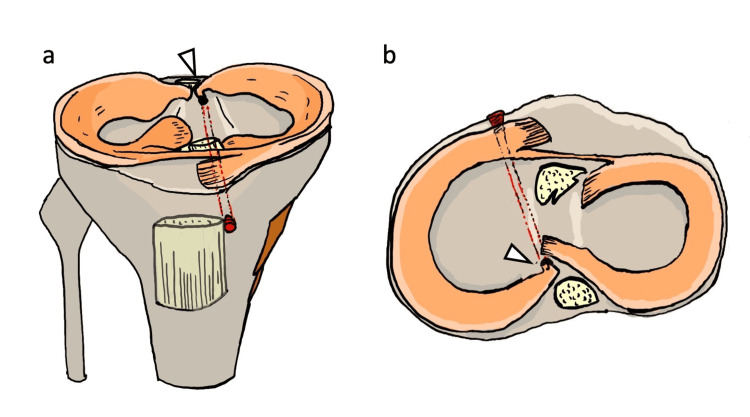
Pull-out repair using a medial tibial tunnel (a) Coronal view of the pull-out repair using a medial tibial tunnel. The white arrowhead indicates a sharp angulation—commonly referred to as the "killer turn"—at the posterior root of the medial meniscus. (b) Axial view of the same configuration. The white arrowhead highlights the acute angle of the posterior root as it exits the tunnel, representing the killer turn. Illustration created by the authors

**Figure 10:**
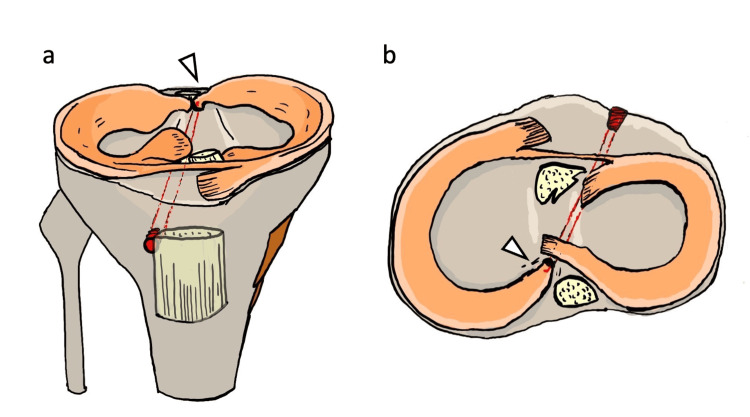
Pull-out repair using an anatomical tibial tunnel (a) Coronal view of the pull-out repair using an anatomical tibial tunnel aligned with the native vector of the medial meniscus posterior root. The white arrowhead indicates a smooth, anatomic trajectory without a killer turn. (b) Axial view of the same anatomical tunnel, again showing an unobstructed path of the posterior root (white arrowhead), avoiding excessive angulation. Illustration created by the authors

In cases combining OWHTO with pull-out repair, interference between the tibial tunnel and the plate screws is a frequent concern, particularly when using the MT approach [21]. In this study, we found that the AT showed minimal interference with screws. Even when contact occurred, more than half of the screw length (mean 58.5%) could still be inserted, whereas MT frequently required either very short screws or left the hole vacant due to direct overlap. This suggests that the AT may offer greater procedural safety and flexibility in hardware placement during OWHTO. Furthermore, the AT reflects the anatomical vector of the MMPR, reducing the so-called “killer turn” and aligning the graft with the native fiber direction (Figures 9, 10). The tunnel orientation also reduces the risk of posterior neurovascular injury during guide pin insertion [22]. Compared to MT, which often deviates medially due to slippage along the tibial border [14], the AT enables more accurate anatomic positioning of the MM attachment site [5,22-24]. Collectively, these findings support AT as a promising alternative to MT when performing pull-out repair in conjunction with OWHTO.

Postoperative changes in tibial tunnel diameter were compared between AT and MT. The MT group showed early postoperative tunnel enlargement followed by gradual narrowing, whereas the AT group demonstrated a tendency toward tunnel diameter reduction from the early phase. Although the mechanism remains unclear, the anatomical alignment of the AT may contribute to reduced micromotion and better tunnel stability. These preliminary findings warrant further investigation.

The AT aligns with the anatomical vector of the MMPR, potentially avoiding the “killer turn” and improving the biomechanical environment for root healing. Compared to MT, AT was associated with less screw interference in OWHTO, and even in cases with overlap, screw insertion was still feasible. Additionally, AT showed favorable changes in tunnel diameter over time. These findings suggest that AT may be a viable alternative when performing pull-out repair in combination with OWHTO. However, AT requires an additional skin incision and careful attention to posterior neurovascular structures. Furthermore, this approach has been applied in a limited number of cases, and long-term clinical outcomes remain unknown. Variations in individual knee anatomy, such as tibial plateau morphology or coronal alignment, may also influence tunnel placement accuracy, highlighting the need for further validation in larger, prospective studies (Table 3).

**Table 3:** Comparison of anatomical tibial tunnel (AT) and medial tibial tunnel (MT) in pull-out repair Values represent the qualitative comparison between AT and MT approaches. Key aspects include the presence of the killer turn, risk of posterior neurovascular injury, medialization of the tibial tunnel, and postoperative changes in tibial tunnel diameter.

	AT	MT
Killer turn	No	Yes
Risk of damaging the posterior neurovascular structure	Low	High
Medialization of the proximal hole of the tibial tunnel	No	Yes
Tibial tunnel diameter	Tendency to decrease from the early postoperative period	May exhibit initial enlargement and subsequently decrease over time

This study has several limitations. First, the MRI vector analysis was performed by a single examiner using a simplified method. Second, the number of clinical cases, particularly those evaluated longitudinally, was small. Third, the drill size used to create the tibial tunnel (4.0 mm or 4.5 mm) was not recorded in each case, which may have influenced the assessment of tunnel diameter changes over time. To address this, relative comparisons using the one-week postoperative diameter as a baseline were adopted. Fourth, other anatomical factors, such as tibial plateau morphology or coronal alignment, were not evaluated, despite their potential influence on tunnel orientation. Finally, the long-term clinical outcomes of AT remain unclear and require further prospective investigation.

## Conclusions

In conclusion, this study elucidated the anatomical vector of the MMPR, enabling three-dimensional capture of the potentiality of pull-out repair. The AT, oriented along the MMPR fibers, showed advantages in reducing the killer turn and promoting early tibial tunnel filling. Additionally, when combined with OWHTO, the AT improved interference issues with screws. These preliminary findings suggest that the AT may offer anatomical and surgical advantages in MMPR pull-out repair; however, further studies with larger sample sizes are needed to confirm these results.
